# Changes in Predominance of Pulsed-Field Gel Electrophoresis Profiles of *Bordetella pertussis* Isolates, United States, 2000–2012

**DOI:** 10.3201/eid2203.151136

**Published:** 2016-03

**Authors:** Pamela K. Cassiday, Tami H. Skoff, Selina Jawahir, M. Lucia Tondella

**Affiliations:** Centers for Disease Control and Prevention, Atlanta, Georgia, USA (P.K. Cassiday, T.H. Skoff, M.L. Tondella);; Minnesota Department of Health, St. Paul, Minnesota, USA (S. Jawahir)

**Keywords:** *Bordetella pertussis*, pulsed-field gel electrophoresis, pertactin, bacteria, respiratory infections, United States, pertussis, whooping cough

## Abstract

To clarify the characteristics of circulating *Bordetella pertussis* isolates, we used pulsed-field gel electrophoresis (PFGE) to analyze 5,262 isolates collected in the United States during 2000–2012. We found 199 PFGE profiles; 5 profiles accounted for 72% of isolates. The most common profile, CDC013, accounted for 35%–46% of isolates tested from 2000–2009; however, the proportion of isolates of this profile rapidly decreased in 2010. Profile CDC237, first seen in 2009, increased rapidly and accounted for 29% of 2012 isolates. No location bias was observed among profiles during 2000–2010, but differences were observed among isolates from different states during 2012. Predominant profiles match those observed in recent European PFGE studies. PFGE profile changes are concurrent with other recent molecular changes in *B. pertussis* and may be contributing to the reemergence of pertussis in the United States. Continued PFGE monitoring is critical for understanding the changing epidemiology of pertussis.

## Abstract 2

These changes are concurrrent with other recent molecular changes and may be contributing to US pertussis reemergence.

Despite high coverage with *Bordetella pertussis* component–containing vaccines, the incidence of reported pertussis has been increasing in the United States, and notable pertussis outbreaks have occurred in recent years (*1*). More than 48,000 pertussis cases were reported in the United States in 2012, the highest number reported since 1955 (*2*). Multiple factors have likely contributed to this increase, including increased recognition of pertussis among the general population, increased diagnosis of healthcare providers, improved diagnostic testing and reporting, and waning immunity from pertussis vaccines (*3*–*5*).

Concerns over adverse reactions after receipt of vaccines containing whole-cell preparations (WCVs) of *B. pertussis* led to development of vaccines with less reactogenicity (*6*). Starting in the 1990s, vaccines with acellular pertussis components (ACVs) began replacing the use of WCVs in the United States, and in 2005 ACVs with a lower concentration of pertussis components (known as Tdap vaccines for their tetanus and diphtheria toxoids and acellular pertussis components) were recommended for the first time as a booster dose among adolescents and adults (*7*,*8*).

Researchers in several countries have used methods such as pulsed-field gel electrophoresis (PFGE), multilocus sequence typing (MLST), and multilocus variable-number tandem-repeat analysis (MLVA) to study the evolution of molecular changes in *B. pertussis* populations over time (*9*–*25*). Recent studies have documented genetic changes in currently circulating *B. pertussis* strains, including shifts in virulence-associated protein phenotypes and predominant molecular types between prevaccine and postvaccine eras. These shifts may also be contributing to the resurgence of pertussis as a result of pathogen adaptation to current pertussis vaccines (*26*–*28*). In addition, recent changes in the epidemiology of pertussis have highlighted an increasing number of cases among older children and adolescents who have been fully vaccinated with ACVs; this increase further supports a role for vaccine pressure in the reemergence of pertussis in the United States (*1*,*3*–*5*,*7*).

PFGE, which can differentiate between individual isolates, has been used to characterize US *B. pertussis* isolates for >15 years (*19*,*29*). Because this method uses the entire genome, PFGE typing is more discriminatory for *B. pertussis* than PCR-based typing methods, such as MLST and MLVA, which analyze the sequences of a few select loci (*22*,*30*). MLST and MLVA typing must be used in tandem to obtain discriminatory power similar to that of PFGE typing (*13*,*30*). 

Our objective was to increase understanding of the reemergence of pertussis and characteristics of circulating *B. pertussis* strains in the United States. To determine the current distribution of circulating PFGE profiles and identify changes in profile distributions over time, we analyzed PFGE profiles of *B. pertussis* isolates collected in the United States during 2000–2012. 

## Materials and Methods

A total of 5,262 isolates were available for testing, collected from 32 states during 2000–2012 (Table). Isolates were identified and collected through Enhanced Pertussis Surveillance (EPS) conducted as part of the Emerging Infections Program Network, through routine state and local health department pertussis surveillance, or during localized outbreaks. Isolates from Massachusetts and Minnesota were characterized by PFGE in their respective state public health laboratories, and tagged image file format images of PFGE gels were sent to the Centers for Disease Control and Prevention (CDC) for analysis; isolates from all other states were sent directly to CDC for PFGE characterization (Table). Because running conditions differed from those of the PFGE method commonly used in Europe, images produced by each method could not be directly compared (*9*,*11*,*12*,*16*–*18*,*23*). An additional set of 5 *B. pertussis* isolates, representing European profiles BpSR3, BpSR5, BpSR10, BpSR11, and BpSR12, was also included in this study with which to compare US isolates with the most commonly circulating PFGE profiles in several European countries during 1998–2009 (*12*).

**Table:** *Bordetella pertussis* isolates by state and year of collection, United States, 2000–2012

State	2000	2001	2002	2003	2004	2005	2006	2007	2008	2009	2010	2011	2012	Total no. (%)
Arizona	52	3	70	56	33	184	12	0	0	0	0	0	1	411 (8)
California	27	10	36	13	7	35	22	1	0	2	38	10	1	202 (4)
Colorado	0	0	0	0	0	0	0	1	2	0	2	0	9	14 (>1)
Connecticut	0	0	0	0	0	0	0	0	0	0	0	7	16	23 (>1)
Delaware	0	0	0	0	0	3	0	0	0	0	0	0	0	3 (>1)
Florida	0	0	0	0	0	0	0	0	0	1	0	6	9	16 (>1)
Georgia	34	13	21	14	13	22	20	3	12	56	4	1	5	218 (4)
Idaho	1	0	1	0	0	0	0	0	0	0	0	0	0	2 (>1)
Illinois	32	27	14	25	0	19	2	11	7	0	0	0	0	137 (3)
Indiana	3	0	0	0	0	0	0	0	0	0	0	0	0	3 (>1)
Kentucky	0	0	0	1	0	0	0	0	0	0	0	0	0	1 (>1)
Maryland	0	0	0	2	0	0	0	0	0	0	0	0	0	2 (>1)
Massachusetts*	227	66	84	178	296	204	194	216	133	79	81	19	0	1,777 (34)
Michigan	0	0	0	0	0	0	0	0	0	0	2	0	0	2 (>1)
Minnesota*	186	98	113	67	83	109	40	30	98	44	29	5	91	993 (19)
Missouri	0	2	1	0	8	0	0	0	0	0	0	1	0	12 (>1)
Nebraska	0	0	0	0	0	3	0	0	0	0	0	0	0	3 (>1)
Nevada	0	0	6	0	0	0	0	0	0	0	0	0	0	6 (>1)
New Jersey	1	1	1	0	0	0	0	0	0	0	0	0	0	3 (>1)
New Mexico	0	0	0	0	0	0	0	8	8	10	0	2	4	32 (1)
New York	29	21	29	73	169	41	0	0	0	0	2	9	53	426 (8)
North Carolina	0	0	0	0	0	0	0	0	11	0	0	0	0	11 (>1)
Ohio	74	72	0	1	1	0	0	0	0	0	0	0	0	148 (3)
Oregon	0	0	0	0	0	0	0	0	0	0	6	19	86	111 (2)
Pennsylvania	0	0	1	2	0	0	0	0	0	3	19	6	0	31 (1)
South Carolina	0	0	0	10	0	2	0	0	3	1	3	0	0	19 (>1)
Tennessee	0	0	0	0	0	0	0	0	0	0	2	0	0	2 (>1)
Texas	11	0	2	0	0	0	0	0	0	0	0	0	0	13 (>1)
Utah	0	1	0	0	0	0	0	0	0	0	0	0	0	1 (>1)
Vermont*	0	0	0	0	0	0	0	18	1	2	1	44	333	399 (8)
Virginia	0	0	0	0	0	0	0	0	1	0	0	0	0	1 (>1)
Washington*	0	0	0	9	0	0	0	0	0	0	0	9	222	240 (5)
Total no. (%)	677 (13)	314 (6)	379 (7)	451 (8)	610 (12)	622 (12)	290 (5)	288 (5)	276 (5)	198 (4)	189 (4)	138 (3)	830 (16)	5,262 (100)

*Isolates from these states were included in the subanalyses presented in Figure 3.

PFGE was performed by using restriction enzyme *Xba*I (*19*), based on the method developed by Gautom et al. (*31*) and similar to that currently used by US state health departments participating in CDC’s PulseNet (http://www.cdc.gov/pulsenet/pathogens/index.html) for the typing of foodborne pathogens (*32*). PFGE patterns were compared with those in a database of *B. pertussis* isolate profiles maintained at CDC, and profiles were assigned on the basis of bands in the 125- to 450-kb range by using BioNumerics software version 5.01 (Applied Maths, Austin, TX, USA).

To identify predominantly circulating profiles, we analyzed overall circulating profiles for each individual year of the study period. Distributions were also compared during 2 shorter periods (2000–2009 and 2010–2012) to assess differences between recent peak years in disease and the remainder of the study period. To assess how states that submitted a large proportion of isolates affected the overall study findings, we performed 2 subanalyses. First, since Massachusetts and Minnesota together contributed >50% of isolates annually during 2000–2010 (Table), we conducted a subanalysis to check for profile local bias by comparing isolates collected from these states during 2000–2010 (Massachusetts, n = 1,758; Minnesota, n = 897), with isolates collected from all other states combined (n = 1,639). A second subanalysis was conducted in which isolates obtained during 2012 from 2 states that experienced substantial statewide epidemics of pertussis, Vermont (n = 333) and Washington (n = 222), were compared with isolates obtained from the other 10 states (n = 275) in 2012 to assess the distribution of circulating PFGE profiles during a period of widespread disease. The χ^2^ test was used for comparison of proportions; p<0.005 was considered significant.

A dendrogram of predominant profiles was created using BioNumerics software to visualize the degree of similarity between identified PFGE patterns. Dendrogram analysis also included the 5 predominant profiles found recently circulating in Europe. Clustering was determined by using unweighted pair group method with arithmetic mean with 1% band tolerance and optimization settings.

To assess changes among the study isolates over time, we calculated genetic diversity overall and by year of isolation using the Simpson Index of Diversity (*33*). CIs were calculated as described by Grundmann et al. (*34*).

## Results

**Figure 1:**
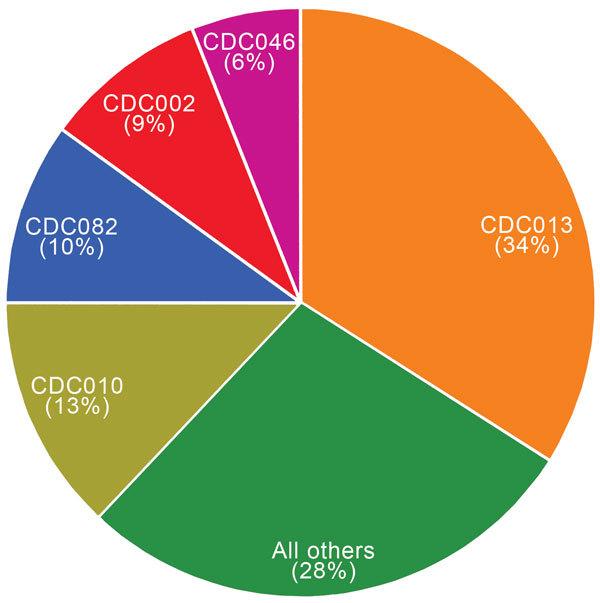
Predominant pulsed-field gel electrophoresis profiles of 5,262 *Bordetella pertussis* isolates, United States, 2000–2012.

**Figure 2:**
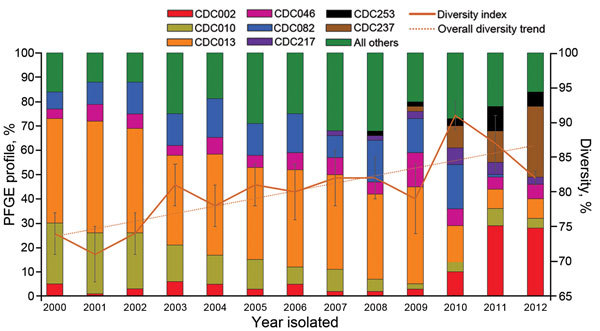
Predominant pulsed-field gel electrophoresis (PFGE) profiles and genetic diversity of 5,262 *Bordetella pertussis* isolates, by year of collection, United States, 2000–2012.

Overall, we identified 199 distinct PFGE profiles among the study isolates. CDC013, CDC010, CDC082, CDC002, and CDC046 were the predominant 5 profiles among our study population and accounted for 72% of all isolates tested (Figure 1). One additional profile, CDC237, accounted for 5% of isolates overall but was only observed in isolates collected in 2009 and subsequently (Figure 2). None of the remaining 193 profiles accounted for >5% of isolates overall.

When we assessed the most predominant profiles by year, we noted differences in the order of predominance beginning in 2010 (Figure 2). CDC013 was consistently predominant during 2000–2009, but CDC082 was predominant among isolates collected in 2010. CDC002 appeared to be fading from circulation during 2000–2009, but it emerged as the most common profile among 2011 isolates. CDC237 and CDC002 predominated in 2012; each accounted for ≈29% of circulating profiles.

When the 5 most predominant profiles were compared in each period, we found CDC013 in 41% of isolates collected during 2000–2009 (35%–46% annually) but only in 9% of isolates analyzed during 2010–2012 (p<0.0001). Prevalence also declined significantly between periods for CDC010 (from 15% to 4.5%, p<0.0001) and CDC082 (from 12% to 3%, p<0.0001). Conversely, profile CDC002, which comprised 4% of isolates in the earlier period, increased to 25% among isolates collected during 2010–2012 (p<0.0001). No changes were observed for profile CDC046, which accounted for a consistent 6% of isolates in each period. In addition, frequency of 3 profiles (CDC217, CDC237, CDC253), each accounting for <1% of isolates during 2000–2009, increased significantly to 4%, 24%, and 6%, respectively, of isolates collected during 2010–2012 (p<0.0001 for all).

**Figure 3:**
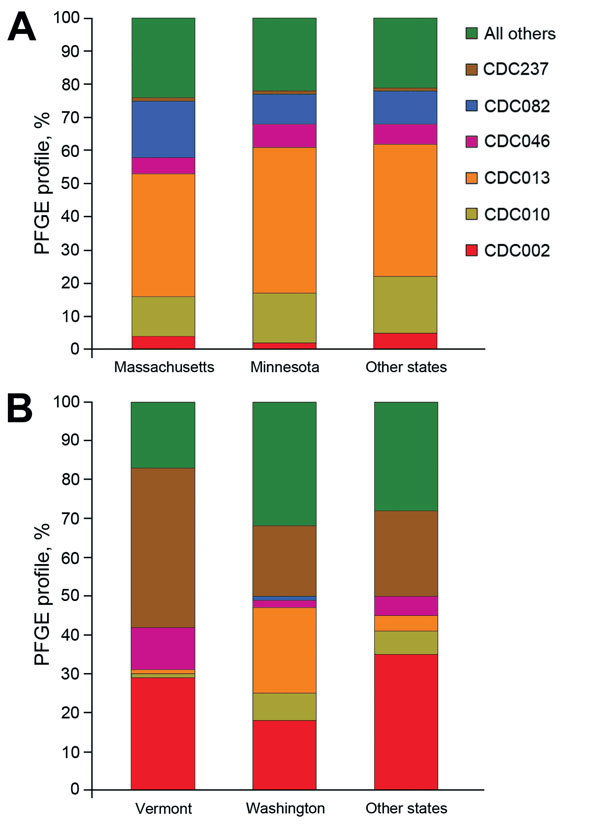
Geographic comparison of predominant pulsed-field gel electrophoresis (PFGE) profiles of *Bordetella pertussis* isolates, United States. A) Predominant profiles in Massachusetts and Minnesota compared with isolates from other states, 2000–2012. B) Predominant profiles in Vermont and Washington compared with isolates from other states, 2012.

When we compared profiles of Massachusetts and Minnesota isolates to isolates of all other states combined during 2000–2010, our subanalysis revealed that CDC013 was the most common PFGE profile among all 3 groups, ranging from 37% to 44% of isolates (Figure 3, panel A). In addition, CDC010 accounted for a similar proportion of isolates across all groups (12%–17%). Whereas a similar proportion of isolates were of profile CDC082 in Minnesota and in all other states combined (9% and 10%, respectively), this profile was found in significantly higher proportion in Massachusetts (17%, p<0.0001), where it was the second most predominant profile. CDC046 accounted for a consistent 5%–7% of isolates from all 3 groups, and CDC002 frequency ranged between from 2% to 5%.

Our subanalysis of the predominant profiles in 2012 isolates from 2 statewide epidemics (Vermont and Washington) and 10 other states combined revealed marked differences between the collections (Figure 3, panel B). CDC013 predominated among Washington isolates at 22%, which was significantly more than the 1% and 4% of isolates with this profile found among isolates from Vermont and combined states, respectively (p<0.0001). In Vermont, a higher percentage of isolates was CDC237 (41%), compared with 18% and 22% of isolates from Washington and the other states combined, respectively (p<0.0001). CDC046 was also found in significantly higher proportion among Vermont isolates (11%) than among those from Washington (1%; p<0.0001), but the proportion among isolates from the other states (5%, p = 0.0098) was not significantly higher. The most predominant profile from the other states combined group was CDC002 (35%), which accounted for a similar proportion of isolates from Vermont (29%, p = 0.0966), but a significantly higher proportion of isolates from Washington (18%, p<0.0001). CDC010 comprised only 1% of isolates from Vermont, significantly fewer than those from Washington (7%, p = 0.0003) and the other states combined (6%, p = 0.0021). CDC082 accounted for <1% of isolates in each group.

**Figure 4:**
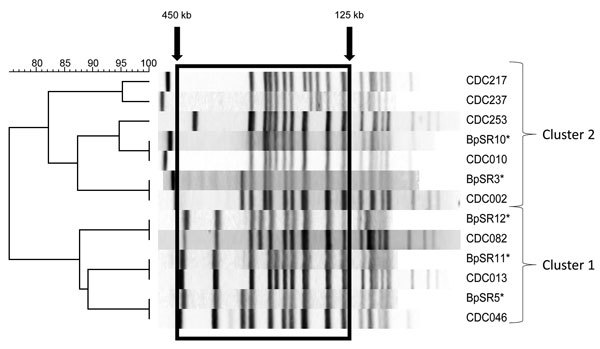
Dendrogram of the predominant *Bordetella pertussis* pulsed-field gell electrophoresis (PFGE) profiles currently circulating in the United States. Clusters were determined by using unweighted pair group method with arithmetic mean (UPGMA) with 1% band tolerance and optimization settings. Box indicates area of band analysis (125 kb–450 kb). *Indicates the predominant *B. pertussis* PFGE profiles currently circulating in Europe, as reported by Advani et al. (*12*). In the area of band analysis, these profiles (BpSR3, BpSR10, BpSR11, BpSR5, and BpSR12) were indistinguishable from US profiles CDC002, CDC010, CDC013, CDC046, and CDC082, respectively. Scale bar indicates percentage similarity.

A dendrogram analysis comparing the 5 most predominant profiles overall in the United States during the study period (CDC013, CDC010, CDC082, CDC002, CDC046) with 3 increasingly circulating profiles (CDC217, CDC237, CDC253) revealed 2 clusters with 75% similarity. Cluster 1 contains profiles CDC013, CDC046, and CDC082, first seen among US isolates collected during 1993–1997. Cluster 2 contains profiles CDC002, CDC010, CDC217, CDC237, and CDC253. CDC002 and CDC010 were first seen among US isolates collected in the 1980s, whereas the other 3 profiles were not seen until 2007. In the area of band analysis used in this study (125 kb–450 kb), the profiles of the 5 predominant strains from Europe, (BpSR3, BpSR10, BpSR11, BpSR5, BpSR12) were indistinguishable from the 5 predominantly circulating US profiles (Figure 4).

Overall genetic diversity among the study isolates was 84%. Genetic diversity ranged from 71% to 82% annually for isolates collected during 2000–2009 before a high of 91% was reached in 2010 (Figure 2). By 2012, diversity decreased slightly to 82%.

## Discussion

Of the 199 *B. pertussis* PFGE profiles observed in this study, a single profile, CDC013, predominated between 2000 and 2009, and 5 profiles comprised most isolates tested from 2000 to 2012. These finding are similar to those previously reported by Hardwick et al., who also found 3 predominant profiles circulating in the United States during 1986–1999 (*19*). Hardwick et al. reported the 3 predominant profiles as CYXXI-010 (now CDC010) (37%), CYXXI-002 (now CDC002) (11%), and CYXXI-013 (now CDC013) (10%), which are still among the currently circulating predominant profiles in the United States. Additionally, 42% of the profiles observed during 1986–1999 were still circulating in the United States during our study. Other studies have also shown that a small number of PFGE profiles usually make up most of the circulating *B. pertussis* strains during outbreak and nonoutbreak periods (*12*,*29*). 

In contrast to the isolates collected during 2000–2009, profile predominance changed rapidly among isolates collected during 2010–2012, and a different profile predominated each year. Rare profiles such as CDC237 quickly became more common than the previously predominant CDC013 profile. Two additional rarely seen profiles, CDC217 and CDC253, which were closely related to the CDC237 profile, also increased significantly in recent years. These findings prompted us to explore possible associations between PFGE profile and other molecular changes that have recently occurred in the organism. Specifically, in recent years, pertactin-deficient *B. pertussis* isolates have rapidly emerged. Pertactin is a key immunogen included in all ACVs currently used in the United States (*21*,*30*), and data have suggested a possible selective advantage of pertactin-deficient mutants among ACV-vaccinated populations (*27*). Using supplemental data, we observed that 87% of 2012 isolates included in our analysis were pertactin-deficient. Of interest, isolates with PFGE profiles CDC002 and CDC237, the most common profiles among 2012 isolates, were significantly more likely to be pertactin-deficient than isolates with profile CDC013, the most common profile during 2000–2009. That these 2 profiles started becoming more predominant in 2010, coinciding with the rapid increase in pertactin-deficient isolates in the United States (*21*), suggests a linkage between the 2 changes. To date, no associations have been reported between pertactin-deficiency and specific PCR-based MLVA or MLST types, which reinforces the discrimatory power of PFGE typing methods and highlights the value of this method for elucidating *B. pertussis* evolution on a more granular level (*24*,*30*).

Although Massachusetts and Minnesota contributed a large proportion of isolates during 2000–2010, we observed similar distributions among 4 of the 5 predominant PFGE profiles when we compared isolates collected in these 2 states to isolates in all other states combined. These results suggest that location bias did not affect the findings. This observation contrasts with results of our subanalysis of 2012 isolates, in which significant differences were noted in the predominant strains found in Vermont, Washington, and all other states combined. This finding could be caused by many factors, including clonal expansion within epidemic areas and, as previously noted, selective advantage of pertactin-deficient *B. pertussis* among vaccinated populations. Exploration of epidemiologic data associated with these isolates may further explain the observed geographic differences in PFGE profiles.

Our analysis showed that the 5 most predominant profiles circulating in several countries in Europe (Denmark, Finland, France, Germany, the Netherlands, Norway, Sweden, United Kingdom) during 1998–2009 (*12*) were also the 5 most predominant profiles circulating in the United States during 2000–2012. The proportion of the most predominant profile in Europe, BpSR11, changed from 25%–30% of 1998–2005 isolates to 13% of 2007–2009 isolates, which was similar to changes observed in the United States equivalent CDC013 profile (41%, 2000–2009, to 9%, 2010–2012). Comparable increases were also noted for CDC002 (4%, 2000–2009, to 25%, 2010–2012) and its European equivalent, BpSR3 (0%, 1998–2005, to 21%, 2007–2009). 

We did not detect a large increase in CDC010 during 2010–2012, such as was reported for BpSR10 during 2007–2009 in Europe, but instead detected a large increase in CDC237, first seen among US isolates in 2009. As shown, CDC010 and CDC0237 are extremely similar and cluster together in our dendrogram (Figure 4), which indicates that they are closely related. Possible explanations for differences in these profiles include a point mutation with the gain of a restriction site in CDC237 isolates or a rearrangement at the chromosomal level (*23*,*35*,*36*). 

Similar to the findings for US isolates, the study in Europe also reported increased diversity (*12*). However, even though circulating PFGE profiles appear to be similar in the United States and Europe, percentages of pertactin-deficient isolates collected during 2010–2014 in European countries are much lower than in the United States, which suggests that even though PFGE changes and pertactin deficiency are occurring simultaneously in the United States, these changes are not necessarily linked and that virulence and transmission of pertussis depend on many factors (*37*,*38*).

A limitation of our study is that the differences in switch times and running time of our PFGE method prevents direct comparison with isolates tested by the European PFGE method (*23*). Our method results in low resolution of 6–8 small (45- to 125-kb) DNA bands, which are not included as part of our analysis, leading to fewer PFGE profiles overall when compared to the European method. However, by using the same PFGE method as in our previous study, we were able to directly compare 6,595 US isolates collected over 77 years. The profile changes observed in the course of the 2 studies have provided useful information for the development of current molecular typing systems and whole-genome sequencing strategies.

Our work reveals that *B. pertussis* PFGE profiles are changing rapidly in the United States, as they are in Europe (*12*). The previously held assumption that *B. pertussis* was a highly clonal organism has been challenged because recent isolates of *B. pertussis* have been shown to be quite variable at the genome level, particularly in those genes that code for ACV components (*21*,*27*,*28*). Although more emphasis is now being placed on molecular typing methods that do not require the availability of isolates, PFGE, in tandem with other typing methods, continues to remain a valuable tool for understanding *B. pertussis* because it can detect a greater level of diversity among circulating strains. 

As we move more toward whole-genome sequencing and rearrangement analysis, PFGE can be useful for strain selection when genetic diversity is a desired parameter for analysis. Having a more complete picture of the molecular evolution of *B. pertussis* through whole-genome methods such as PFGE and sequencing may provide key data to help guide development of the next generation of pertussis vaccines or, in the near term, inform us on ways to more effectively use existing vaccines. Continued monitoring of the molecular epidemiology of circulating *B. pertussis* continues to be critical for understanding the changing epidemiology of the disease both in the United States and abroad to optimize current prevention and control strategies.
